# Risk factor analysis of thymoma resection and its value in guiding clinical treatment

**DOI:** 10.1002/cam4.6043

**Published:** 2023-05-08

**Authors:** Xin Du, Jian Cui, Xin‐tao Yu, Lei Yu

**Affiliations:** ^1^ Department of Thoracic Surgery Beijing Tongren Hospital, Capital Medical University Beijing China

**Keywords:** clinicopathologic characteristics, prognosis, progression‐free survival (PFS), thymoma, treatment

## Abstract

**Background:**

In this study, relationships between clinicopathologic characteristics and progression‐free survival (PFS) of patients after thymomectomy were analyzed to provide valuable suggestions regarding the treatment of thymoma.

**Methods:**

Data from 187 thymoma patients undergoing surgery at Beijing Tongren Hospital between January 1, 2006, and December 31, 2015, were retrospectively reviewed. We explored the risk factors for PFS among sex, age, thymoma‐associated MG, completeness of resection, histologic type and TNM stage, and investigated their interrelationship.

**Results:**

Among the 187 patients, 18 patients (9.63%) had tumor recurrence/metastasis, and all of whom had in situ recurrence or pleural metastasis, and most of them (10 of 18 patients) had MG symptoms that reappeared or were aggravated. Fifteen patients (8.02%) died, and myasthenic crisis was a leading cause. Based on Cox regression analysis, only age (HR = 3.16; 95% CI: 1.44–6.91; *p* = 0.004) and the completeness of resection (HR = 9.03; 95% CI: 2.58–31.55; *p* = 0.001) were independent risk factors for PFS. Furthermore, we found that the completeness of resection was related to the histologic type (*p* = 0.009) and TNM stage (*p* < 0.001) by Fisher's exact test.

**Conclusions:**

The results of this cohort study remind us that we should pay attention to the reappearance or aggravation of MG after thymoma resection, because it is the leading cause of death and may indicate tumor progression. Furthermore, completeness of resection was related to the histologic type and TNM stage, but it was the independent risk factors of thymoma. Therefore, R0 resection is critical to the prognosis of thymoma.

## 1 INTRODUCTION

Thymoma is the most common primary anterior mediastinal mass derived from the thymic epithelium. Its incidence is approximately 1.5/1,000,000 person‐years.^1^, ^2^, ^3^ In general, approximately 30%–50% thymoma patients suffer from paraneoplastic syndrome, and, of these, myasthenia gravis (MG) is the most common.^4^, ^5^ In the 2021 WHO Classification of Tumors of the Thymus and Mediastinum, thymoma is mainly divided into five types (A, AB, B1, B2 and B3) based on the rate of nonmalignant‐appearing thymic epithelial cells and proportions of lymphocytes.^6^ The latest TNM classification system classifies thymoma into I‐IV stages mainly according to the local extension of the primary tumor, lymph node involvement and metastasis via clinical and histopathologic examination.^6^

Currently, radiotherapy is recommended for incompletely resected thymomas, but there are still concerns about radiotherapy for completely resected stage II‐III thymomas.^7^, ^8^ Chemotherapy is used only for neoadjuvant treatment of locally advanced tumors and palliative therapy of stage IV thymomas.^9^, ^10^, ^11^ Immunotherapy and targeted therapy are not recommended for thymoma patients because of concerns about immune‐related events.^12^, ^13^ Surgery (total thymectomy and complete excision of the tumor) is still the mainstay of treatment for thymoma patients.^11^, ^14^, ^15^ However, there are few comprehensive studies on preoperative, intraoperative and postoperative management strategies for thymoma. The present retrospective cohort study aimed to analyze the risk factors for PFS after thymomectomy and provide valuable suggestions for the whole‐course treatment of thymoma.

## 2 METHODS

### 2.1 Inclusion and exclusion criteria

This was a retrospective, single‐center study of thymoma patients undergoing surgery at Beijing Tongren Hospital (Myasthenia Gravis & Thymoma Diagnosis and Treatment Center, Capital Medical University) between January 1, 2006, and December 31, 2015. All clinicopathological data were collected from clinical database of Beijing Tongren Hospital and the included patients were consecutive. The inclusion criteria were as follows: (1) no chemotherapy or radiation therapy was administered before surgery; (2) surgery, including radical or palliative resection, was performed; and (3) a postoperative pathological diagnosis of thymoma was made by two independent pathologists. The exclusion criteria were as follows: (1) the presence of other malignant or borderline tumors; and (2) IVB patients who are unfit for surgery. The study was approved by the Ethics Review Board of Beijing Tongren Hospital, and the requirement for informed consent was waived (TRECKY2021‐175).

### 2.2 Surgical approach and observation index

Radical thymectomy includes the resection of the thymoma, thymus, anterior mediastinal adipose tissue around the thymus and the involved adjacent organ and tissues. For patients with pleural or pericardial metastasis (IVA stage), we performed intrapleural perfusion thermo‐chemotherapy treatment during operation. Beyond patients with stage I and complete resection (R0), our center recommended postoperative adjuvant radiotherapy to reduce the risk of tumor recurrence.^16^, ^17^ Due to the long survival of thymoma patients, and stage IVA patients were include in this cohort study, progression‐free survival (PFS) was selected as the most appropriate prognostic index in this study. Referring to previous studies on the prognosis of thymoma, we selected the following data as the indicators affecting PFS: sex, age, thymoma‐associated MG, completeness of resection, histologic type and TNM stage. All the data were collected from clinical database of Beijing Tongren Hospital.

### 2.3 Follow‐up

After surgery, chest CT examinations were reassessed at 6‐month intervals for the first 2 years and then annually thereafter.^18^ Follow‐up was performed through outpatient visits or telephone follow‐up every 6 months. The last follow‐up date was December 31, 2020. During follow‐up, we recorded the survival status, tumor progression time, time of death and reasons for death. The endpoint was PFS, which was defined as the time (months) from operation to tumor progression or death from any cause.

### 2.4 Statistical analysis

Continuous quantitative data are presented as the mean ± standard deviation (SD) and were analyzed with a *t* test. Survival analysis was conducted through the Kaplan–Meier method and analyzed by the log‐rank test. Univariate and multivariate Cox regression were used for risk factor analysis of PFS, and the data are presented as hazard ratios (HR) and 95% CIs. Fisher's exact test was used to evaluate the correlation of risk factors. Statistics were performed using SPSS v.26.0, and the significance was set at two‐tailed *p* < 0.05.

## 3 RESULTS

### 3.1 Patient characteristics

According to the inclusion and exclusion criteria, a total of 187 thymoma patients undergoing surgery at Beijing Tongren Hospital between January 1, 2006, and December 31, 2015, were enrolled in this study. The median follow‐up time was 102 months (range, 62–175 months). The basic characteristics of the 187 included patients are listed in Table 1. Furthermore, 89.29% of patients with stage III underwent R0 resection (complete resection). All the stage IV patients had pleural or pericardial metastasis without lymph node or distant metastasis.

**TABLE 1:** The basic characteristics of patients.

Characteristics	*n*	Percentage (%)
Total	187	100
Age (year)		
Mean ± SD	50.3 ± 12.2	‐
Sex		
Female	98	52.41
Male	89	47.59
Thymoma‐associated MG		
MG	130	69.52
Non‐MG	57	30.48
Type of resection		
Thoracoscopic surgery	143	76.47
Median sternotomy	44	23.53
Completeness of resection^a^		
R0	165	88.24
R1	2	1.07
R2	20	10.70
Histologic type		
A	6	3.21
AB	43	22.99
B1	31	16.58
B2	69	36.90
B3	38	20.32
TNM stage		
I	96	51.34
II	19	10.16
III	56	29.95
IV	16	8.56

^a^ The completeness of resection was classified as R0 = complete resection, R1 = microscopic residual disease infiltrating resection margins and R2 = macroscopic residual disease.

### 3.2 Survival analysis

**FIGURE 1:**
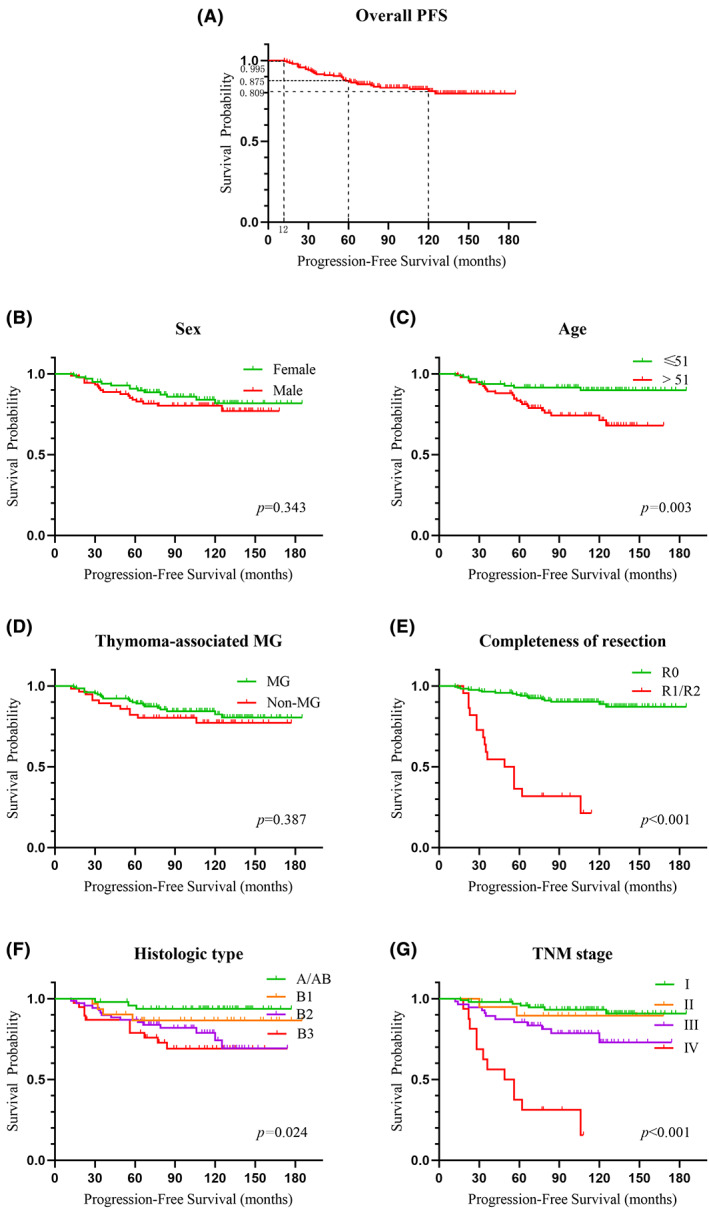
In Panel (A) the Kaplan–Meier survival estimate curve for the overall PFS shown that the 1‐, 5–10‐year PFS rate were 99.5%, 87.5%, and 80.9%, respectively. In Panel (B–G) the Kaplan–Meier curves were drawn according to sex, age, thymoma‐associated MG, completeness of resection, histologic type and TNM stage, respectively.

As of December 31, 2020, 18 patients (9.63%) experienced tumor recurrence/metastasis, and 15 patients (8.02%) had died. All the 18 patients with recurrence/metastasis had in situ recurrence or pleural metastasis, and most of them (10 of 18 patients) had MG symptoms that reappeared or were aggravated. Among the 15 patients who had died, 5 deaths were due to myasthenic crisis (one patient even had no symptoms of MG before surgery), and 10 deaths were due to other diseases. A total of 8 (4.28%) patients were lost to follow‐up. The mean age was 50.3 ± 12.2 years, and the age was grouped by median age of 51. The mean PFS was 158.70 months (95% CI: 150.54–166.86), and the 1‐, 5‐ and 10‐year PFS rates were 99.5%, 87.5%, and 80.9%, respectively (Figure 1A).

By plotting the survival curves with the Kaplan–Meier method and analyzing these data with the log‐rank test, we found that sex (χ^2^ = 0.90; *p* = 0.343) and thymoma‐associated MG (χ^2^ = 0.75; *p* = 0.387) had no effects on PFS, while age (χ^2^ = 8.98; *p* = 0.003), the completeness of resection (χ^2^ = 80.77; *p* < 0.001), histologic type (χ^2^ = 9.44; *p* = 0.024) and TNM stage (χ^2^ = 68.00; *p* < 0.001) had significant effects on PFS (Figure 1B–G).

### 3.3 Risk factor analysis

Univariate Cox regression analysis identified that age (HR = 3.05; 95% CI: 1.41–6.57; *p* = 0.004), the completeness of resection (HR = 13.01; 95% CI: 6.35–26.67; *p* < 0.001), histologic type (*p* = 0.045) and TNM stage (*p* < 0.001) were adverse prognostic factors associated with PFS, but sex (HR = 1.39; 95% CI: 0.70–2.76; *p* = 0.346) and thymoma‐associated MG (HR = 1.37; 95% CI: 0.67–2.78; *p* = 0.389) were not prognostic factors of PFS (Table 2).

**TABLE 2:** Univariate and multivariate Cox regression analysis of PFS.

Variable	Univariate analysis				Multivariate analysis		
	10‐year PFS (%)	HR	95% CI	*p*	HR	95% CI	*p*
Sex				0.346			
Female	81.6	1	–				
Male	80.2	1.39	0.70–2.76				
Age				0.004			0.004
≤51‐year	89.8	1	–		1	–	
>51‐year	71.2	3.05	1.41–6.57		3.16	1.44–6.91	
Thymoma‐associated MG				0.389			
MG	82.4	1	–				
Non‐MG	77.1	1.37	0.67–2.78				
Completeness of resection				<0.001			0.001
R0	88.7	1	–		1	–	
R1/R2	21.2	13.01	6.35–26.67		9.03	2.58–31.55	
Histologic type				0.045			0.611
A/AB	93.6	1	–		1	–	
B1	86.6	2.31	0.52–10.31		2.77	0.60–12.80	
B2	74.2	4.02	1.16–13.89		2.00	0.52–7.66	
B3	69.1	5.63	1.57–20.21		2.16	0.54–8.72	
TNM stage				<0.001			0.731
I	93.2	1	–		1	–	
II	89.5	1.43	0.30–6.86		1.32	0.27–6.43	
III	72.9	3.44	1.35–8.78		1.92	0.62–5.96	
IV	15.6	19.57	7.49–51.14		1.81	0.36–9.08	

Abbreviations: 95% CI, 95% confidence interval; HR, hazard ratio; PFS, progression‐free survival.

The covariates with a *p* value of less than 0.1 in the univariate Cox regression analysis were included in the multivariate Cox regression analysis. The results indicated that only age (HR = 3.16; 95% CI: 1.44–6.91; *p* = 0.004) and the completeness of resection (HR = 9.03; 95% CI: 2.58–31.55; *p* = 0.001) were independent risk factors, while histologic type (*p* = 0.611) and TNM stage (*p* = 0.731) were not the independent risk factors of PFS (Table 2).

Analysis of the correlation among the completeness of resection, histologic type and TNM stage by Fisher's exact test the results showed that completeness of resection was related to histologic type (*p* = 0.009) and TNM stage (*p* < 0.001).

## 4 COMMENT

Due to the limited research on the molecular mechanisms of thymoma, there is currently no prognostic biomarker of this disease. Therefore, it is important to determine the clinicopathologic characteristics that can predict the prognosis of thymoma to guide clinical treatment. In addition, there are relatively few studies on the prognostic factors of thymomas. The previous research indicated that the factors affecting the prognosis of thymoma mainly include sex, age, thymoma‐associated MG, the completeness of resection, histologic type and TNM stage, but the results have varied across different studies.^3^, ^19^, ^20^, ^21^, ^22^, ^23^, ^24^, ^25^ In this study, we conducted a multifactorial analysis of the above clinicopathologic characteristics to identify the independent risk factors for PFS after thymoma resection in order to guide the clinical treatment.

The results showed that histological type and TNM stage affected prognosis but were not independent risk factors for PFS. However, both factors were associated with the completeness of resection, which was one of the two independent risk factors for PFS. Therefore, R0 resection is critical to the prognosis of thymoma, even if the major thoracic structure requires resection and reconstruction in patients with stage III. The view is the same as that of Prof. Deterbeck.^11^ For another independent risk factor, the increase of age will inevitably lead to the increase of basic diseases and affected the prognosis. But one thing is sure: the stage of the tumor will progress over time, which increases the possibility of incomplete resection. Therefore, early detection and early surgery are still vital for the prognosis of thymoma.

In this cohort, none of the patients presented with lymph node metastasis or distant metastasis, and all the patients with recurrence/metastasis had in situ recurrence or pleural metastasis. Therefore, local treatment is the key to the prognosis of thymoma patients. For patients with incomplete tumor resection, intrapleural perfusion thermo‐chemotherapy treatment and postoperative local radiotherapy may prevent tumor progression and metastasis. For patients with complete resection, repeated flushing in the thorax also minimizes the risk of implantation metastasis of shed tumor cells.

In our study, 5/15 (33.33%) of the deaths were attributed to myasthenic crisis, which was the major cause of death related to thymoma in this cohort. Almost all the patients who died from myasthenic crisis occurred outside the hospital due to a lack of ventilator support. Thus, we should pay attention to the occurrence of myasthenic crisis after surgery, even if the patients did not have MG before. Furthermore, we also found that MG symptoms reappeared or were aggravated in most patients with tumor progression or metastasis (10 of 18 patients). Therefore, the reappearance or aggravation of MG in patients after thymoma surgery should prompt clinicians to be vigilant in identifying tumor progression. In this study, MG was the leading cause of death, but the results demonstrated that thymoma‐associated MG had no significant effects on PFS. Interestingly, the proportion of patients with MG in this cohort was 130/187 (69.52%), which was significantly higher than the average of 30%–50%.^26^ Perhaps the combination with MG enable patients to detect thymoma at an early stage, leading to no significant difference in the overall prognosis of patients with MG and those without MG.

There are some limitations of this study. This is a single‐center retrospective study that inevitably has selection bias. This was especially true in regard to the study population and the choice of treatment modality. Furthermore, the effectiveness of local treatment, such us intrapleural perfusion thermo‐chemotherapy treatment and postoperative local radiotherapy, need prospective studies to further prove. However, to make the results more reliable, we excluded the effects of subjective treatment modality and only studied objective indicators, with nearly 10 years of follow‐up.

## 5 CONCLUSIONS

Based on the research results, this study proposes the following suggestions to guide the clinical treatment of thymoma. First, regular chest CT examination, early detection and early surgery are vital for the prognosis of thymoma. Even if major thoracic structures need resection and reconstruction, such as in patients with stage III, R0 resection is critical to the prognosis of thymoma patients. In addition, local treatment, prevention and control of myasthenic crisis may be critical to the prognosis of thymoma. After surgery, regular chest CT examination and the monitoring of MG symptom progression will contribute to the early detection of tumor progression.

## AUTHOR CONTRIBUTIONS

**Xin Du:** Conceptualization (lead); formal analysis (lead); funding acquisition (lead); investigation (lead); methodology (lead); resources (lead); software (lead); validation (lead); visualization (equal); writing – original draft (lead). **Jian Cui:** Conceptualization (equal); formal analysis (equal); investigation (equal); methodology (equal); software (equal); validation (equal); visualization (equal). **Xin‐tao Yu:** Investigation (equal); methodology (equal); software (equal); visualization (equal). **lei yu:** Data curation (equal); funding acquisition (equal); project administration (lead); resources (equal); supervision (equal); writing – review and editing (lead).

## FUNDING INFORMATION

This work was supported by Beijing Association for Science and Technology's "Golden‐Bridge Seed Funding Program" (ZZ22006) and the Hospital Founding of Beijing Tongren Hospital (2021YJJPY011).

## CONFLICT OF INTEREST STATEMENT

The authors declare that they have no known competing financial interests or personal relationships that could have appeared to influence the work reported in this paper.

## ETHICAL APPROVAL

Written informed consent was obtained from the individual(s) for the publication of any potentially identifiable images or data included in this article. Institutional Review Board (IRB) number: TRECKY2021‐175.

## Data Availability

The original contributions presented in the study are included in the article material, further inquiries can be directed to the corresponding author.
